# Immune Reconstitution Inflammatory Syndrome in Acquired Immune Deficiency Syndrome related to Cryptococcal Meningoencephalitis

**DOI:** 10.1177/2324709614533951

**Published:** 2014-04-30

**Authors:** Ji Young Park, Min Jeong Kim

**Affiliations:** 1 Kosin University College of Medicine, Busan, Korea

**Keywords:** highly active antiretroviral therapy, human immunodeficiency virus, immune reconstitution inflammatory syndrome

## Abstract

*Background*. Highly active antiretroviral therapy (HAART) has contributed to reducing the occurrence of opportunistic infections and mortality in human immunodeficiency virus (HIV) infected patients. However, a paradoxical worsening of clinical signs and symptoms among patients during HAART may occur. Immune reconstitution inflammatory syndrome (IRIS) is described as a paradoxical deterioration of clinical status on initiation of HAART in patients with HIV infection. *Case Report*. We describe the case of a 41-year-old man with opportunistic cryptococcal encephalitis who exhibited neurological and radiological deterioration during the course of HAART. A diagnosis of central nervous system (CNS)-IRIS was based on a decrease of HIV-RNA viral load greater than 1 log, with an increase in CD4^+^ T-cell count from baseline. *Conclusions*. Differential diagnosis of this paradoxical deterioration in clinical and neurological status from overwhelming opportunistic infection is important; it enables an avoidance of unnecessary diagnostic procedures and proper management of this HIV-associated CNS disorder.

## Introduction

Immune reconstitution inflammatory syndrome (IRIS) is a potentially life-threatening condition that occurs in a subset of positive human immunodeficiency virus (HIV) patients following initiation of highly active antiretroviral therapy (HAART).^1^ It is characterized by paradoxical worsening of the patient’s clinical condition attributable to recovery of the immune system following introduction of HAART. Rarely IRIS may affect the central nervous system (CNS), especially in the setting of AIDS-related opportunistic infections such as tuberculosis, cryptococcal infection, cytomegalovirus infection, and progressive multifocal leukoencephalopathy (PML).^2,3^ We describe an interesting case of CNS-IRIS in a HIV-positive cryptococcal encephalitis patient, who developed deteriorated neurologic symptoms and as seen by brain imaging after HAART.

## Case

**Figure 1.**
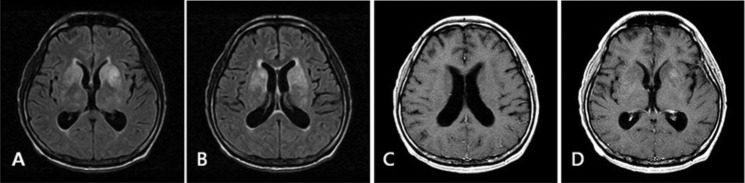
Axial FLAIR (A and B) and T1WI postcontrast (C and D) imaging show hyperintense signal in both the basal ganglia and thalamus with focal enhancement on the left putamen.

A 41-year-old male was admitted to our hospital on January 2012 due to a 3-week history of severe headache, cold sweating, and blurred vision. He had been diagnosed with acquired immunodeficiency syndrome (AIDS) 11 years prior to admission but had not taken any antiretroviral agents. At admission, neurologic examination revealed decreased visual acuity of the right eye, bilateral 6 nerve palsy, and stiff neck. Clusters of tiny T2 hyperintense foci in the bilateral basal ganglia and thalamus were evident in the brain magnetic resonance imaging (MRI), with focal abnormal enhancement lesions in the left putamen (see Figure 1). Lumbar puncture on the day of admission revealed an increased opening pressure (>29 cm H_2_O), 1 cell/mm^3^ of white blood cell with normal protein and decreased glucose concentrations. Cerebrospinal fluid (CSF) cryptococcal antigen was 1:32, and *Cryptococcus neoformans* was cultured from the CSF and blood. The initial CD4^+^ T-cell count was 14 cells/µL with a plasma HIV viral load of 1 420 000 copies/mL. He was treated with amphotericin B lipid complex. Persistent high CSF opening pressures (>60 cm H_2_O) indicated a deterioration in clinical course. He received therapeutic lumbar punctures daily or every other day, and his headache gradually improved over a period of 2 months. Simultaneous with the antifungal treatment, HAART was initiated with lopinavir, ritonavir, zidovudine, and lamivudine. One month after initiation of HAART, antiretroviral agents were switched to efavirenz, lamivudine, and abacavir due to the development of pancytopenia. He responded well to HAART with an increased CD4^+^ T-cell count of 158 cells/µL and plasma HIV viral loads were 123 copies/mL at 3 months after initiation of therapy.

**Figure 2.**
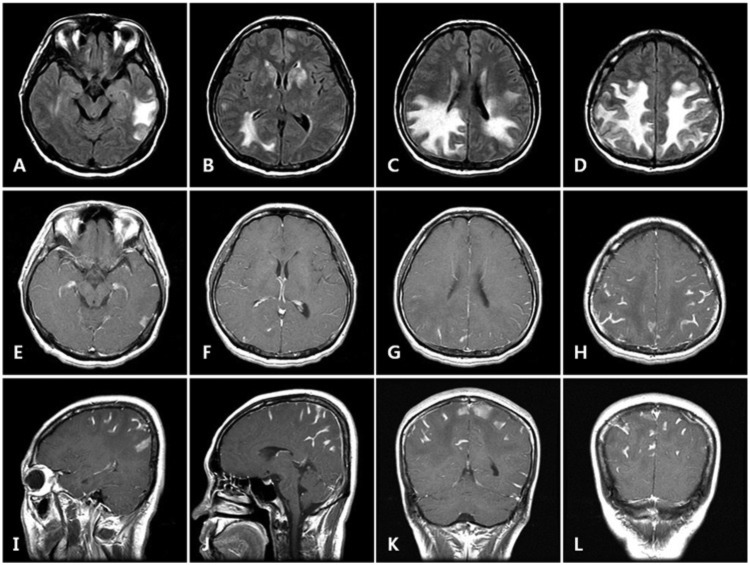
Axial FLAIR (A-D) imaging shows confluent symmetrical hyperintensities in parietal, left temporal, and right occipital white matters. Axial, saggital, and coronal T1WI postcontrast (E-L) imaging shows extensive leptomeningeal enhancement of parietal, temporal, and occipital regions.

**Figure 3.**
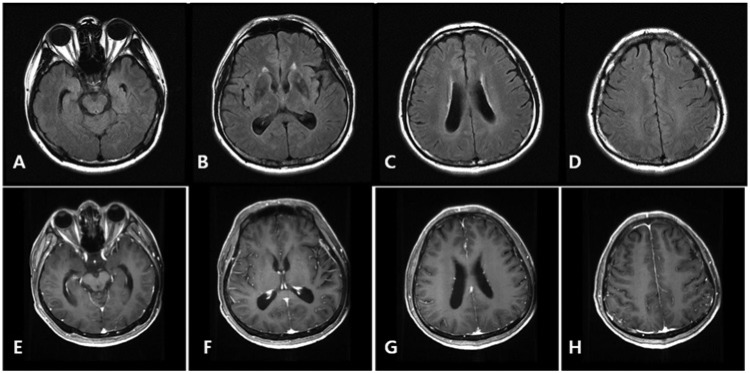
Follow-up axial FLAIR (A-D) and T1WI postcontrast (E-H) imaging show subtle focal hyperintensities without mass effect. No abnormal enhancing lesions are seen in parenchymes but asymmetric focal prominent leptomeningeal enhancement in right parietal cortex is noted.

Five months later, in June 2012, he presented with slurred speech and general weakness. There was no fever and hypertension. Neurologic examination revealed mild dysarthria and clumsiness in both hands. Lumbar puncture was performed and found to be unremarkable with CSF cryptococcal antigen being negative. Epstein–Barr virus, herpes simplex virus 1 and 2, cytomegalovirus, and varicellar zoster virus could not be detected by polymerase chain reaction (PCR), and toxoplasma antibody was not detected. In 2 consecutive CSF studies, JC virus PCR was also negative. Brain MRI demonstrated improved patchy T2 hyperintensities in the BG and thalami, but confluent symmetrical T2 hyperintensities were present in both parietotemporal and right occipital white matter. In contrast enhancement images, extensive leptomeningeal enhancement of both parietotemporal regions was seen (Figure 2). At this stage, CD4^+^ T-cell count was 221 cells/µL and HIV viral load was 47 copies/mL. The patient was diagnosed with IRIS and treated with intravenous methylprednisolone for 5 days. His antiretroviral treatment regimen was maintained. The neurologic abnormalities gradually improved over the next year, and follow-up MRI showed marked improvement (Figure 3).

## Discussion

Neurologic opportunistic infections of HIV infection are important causes of morbidity and mortality. But since the introduction of HAART, there has been a decrease in the incidence of opportunistic infection. IRIS was announced as a paradoxical deterioration in clinical status attributable to the recovery of the immune system during HAART in HIV-infected patients.^1-3^ Diagnostic criteria have not yet been defined, but CNS-IRIS should be suspected when patients present with a rapid deterioration of clinical and neurological status following initiation of HAART; decrease of HIV-RNA viral load greater than 1 log with increase in CD4^+^ T-cell count from baseline; clinical, laboratory, and radiological signs and symptoms consistent with inflammation; and a lack of correlation between symptoms, newly acquired infection, and a previously present opportunistic infection or drug toxicity.^4,5^ IRIS occurs in approximately 20% to 30% of individuals treated with HAART.^1,2^ The majority of cases occur within the first 60 days of initiating HAART; however, it may occur as late as 2 years.^1^

Our patient showed a clinical decline as seen with brain images. The history and clinical presentation involved a broad differential diagnoses including CNS opportunistic infections, PML, acute demyelinating encephalomyelitis (ADEM), reversible posterior leukoencephalopathy, cerebrovascular accident, CNS primary malignancies, autoimmune diseases, as well as drug toxicities. Although a definitive histopathological diagnosis could not be made, our patient met the proposed clinical and laboratory criteria used in defining the diagnosis of CNS-IRIS.^6-8^ The extensive negative microbiological workup in CSF and serum and decrease of HIV-RNA viral load with increase in CD4^+^ T-cell count from very low baseline suggested the possibility of IRIS. We intended a differential diagnosis of PML initially, but negative serum and CSF JC virus PCR in 2 consecutive examinations and brain MRI with contrast, which showed marked meningeal enhancement, alluded to IRIS rather than PML. As for the possible contribution of altered immune response in the HIV-infected patient, we cannot disregard the diagnosis of ADEM. But the possibility of ADEM was thought to be low, because ADEM typically begins within 2 days to 4 weeks after an antigenic challenge, and MRI findings of ADEM are typically asymmetric widespread patchy areas within the white matter and deep gray matter.^9^

The pathophysiology of IRIS remains unclear. On brain biopsy or postmortem examinations, T-lymphocytes and macrophages were found predominantly in the perivascular spaces and the parenchyma, and it has been suggested that T-lymphocytes are associated with pathogenesis of IRIS. The proposed theory of the development of IRIS is based on the vigorous tissue-specific immune reaction to remaining live or dead antigens and a loss of natural immunological regulatory pathways.^5^ During the immunosuppressed phase of HIV infection, CD4^+^ T-cell populations are present at very low absolute numbers because increased viral load restricts the CD4^+^ T-cells to the circulation of target organs. Following the HAART, a rapid decline in viral load triggers redistribution of CD4^+^ T-cells to the peripheral circulation and enhances the ability of antigen presenting cells (APC) to phagocytose.^5^ This results in immune activation of IL-2 release and exuberant inflammatory responses.

Although HAART and the immune reconstitution are considered causes of IRIS, stopping HAART is not recommended. The regrowth of HIV viral replication will cause aggravation of underlying opportunistic infection. More debatable is the use of systemic corticosteroids due to lack of clinical trials to establish the risks and benefits of initiating an immune-suppressive therapy in immune deficient patients. Some reports have suggested that steroids reduce neurologic worsening, radiologic evidence of swelling, mass effect, or brain edema.^10^ Optimal steroid therapy is not established, but generally has been used safely in immunocompromised patients at low dosages for durations of up to 8 weeks. It has been suggested to tailor the dose and duration of systemic corticosteroids according to the clinical presentation.^10-12^

In conclusion, CNS-IRIS is a not an uncommon and life-threatening disorder induced by initiation of HAART and reconstitution of immune functions. Differences between the paradoxical deterioration of clinical and neurological status from overwhelming opportunistic infections are the recovery of CD 4^+^ T-cell count and rapid decline in viral load. As underdiagnosis could contribute to high mortality, knowledge and prompt recognition of the clinical signs and symptoms is most important; it enables an avoidance of unnecessary diagnostic procedures and proper management of this HIV-associated CNS disorder.

## References

[1] Johnson T Nath A Immune reconstitution inflammatory syndrome and the central nervous system. Curr Opin Neurol. 2011;24:284-290. 21499099 10.1097/WCO.0b013e328346be57

[2] French MA Disorders of immune reconstitution in patients with HIV infection responding to antiretroviral therapy. Curr HIV/AIDS Rep. 2007;4:16-21. 17338856 10.1007/s11904-007-0003-z

[3] Müller M Wandel S Colebunders R Attia S Furrer H Egger M IeDEA Southern and Central Africa. Immune reconstitution inflammatory syndrome in patients starting antiretroviral therapy for HIV infection: a systematic review and meta-analysis. Lancet Infect Dis. 2010;10:251-261. 20334848 10.1016/S1473-3099(10)70026-8 PMC4183458

[4] McCombe JA Auer RN Maingat FG Houston S Gill MJ Power C Neurologic immune reconstitution inflammatory syndrome in HIV/AIDS: outcome and epidemiology. Neurology. 2009;72:835-841. 19255411 10.1212/01.wnl.0000343854.80344.69

[5] Shelburne SA 3rd Darcourt J White AC Jr The role of immune reconstitution inflammatory syndrome in AIDS-related *Cryptococcus neoformans* disease in the era of highly active antiretroviral therapy. Clin Infect Dis. 2005;40:1049-1052. 15825000 10.1086/428618

[6] Yoon JH Bang OY Kim HS Progressive multifocal leukoencephalopathy in AIDS: proton MR spectroscopy patterns of asynchronous lesions confirmed by serial diffusion-weighted imaging and apparent diffusion coefficient mapping. J Clin Neurol. 2007;3:200-203. 19513133 10.3988/jcn.2007.3.4.200 PMC2686949

[7] Martin-Blondel G Delobel P Blancher A Pathogenesis of the immune reconstitution inflammatory syndrome affecting the central nervous system in patients infected with HIV. Brain. 2011;134:928-946. 21247931 10.1093/brain/awq365

[8] Mori S Levin P A brief review of potential mechanisms of immune reconstitution inflammatory syndrome in HIV following antiretroviral therapy. Int J STD AIDS. 2009;20:447-452. 19541884 10.1258/ijsa.2009.008521

[9] Tenembaum S Chitnis T Ness J Hahn JS; International Pediatric MS Study Group. Acute disseminated encephalomyelitis. Neurology. 2007;68(suppl 2):S23-S36. 17438235 10.1212/01.wnl.0000259404.51352.7f

[10] Tan K Roda R Ostrow L McArthur J Nath A PML-IRIS in patients with HIV infection: clinical manifestations and treatment with steroids. Neurology. 2009;72:1458-1464. 19129505 10.1212/01.wnl.0000343510.08643.74 PMC2677476

[11] Martinez JV Mazziotti JV Efron ED Immune reconstitution inflammatory syndrome associated with PML in AIDS: a treatable disorder. Neurology. 2006;67:1692-1694. 17101910 10.1212/01.wnl.0000242728.26433.12

[12] McComsey GA Whalen CC Mawhorter SD Placebo-controlled trial of prednisone in advanced HIV-1 infection. AIDS. 2001;15:321-327. 11273211 10.1097/00002030-200102160-00004

